# Foraging as an ethological framework for neuroscience

**DOI:** 10.1016/j.tins.2025.08.006

**Published:** 2025-11

**Authors:** Laura L. Grima, Hannah Haberkern, Rishika Mohanta, Mai M. Morimoto, Adithya E. Rajagopalan, Emma V. Scholey

**Affiliations:** 1 Janelia Research Campus, Howard Hughes Medical Institute, Ashburn, VA 20147, USA; 2 Department of Behavioral Physiology and Sociobiology, Biocenter, University of Würzburg, 97074 Würzburg, Germany; 3 Laboratory of Neurophysiology and Behavior, Laboratory of Social Evolution and Behavior, The Rockefeller University, New York, NY, USA; 4 Howard Hughes Medical Institute, New York, NY 10065, USA; 5 Department of Life Sciences, Imperial College London, London, UK; 6 Center for Neural Science, New York University, New York, NY, USA; 7 Centre for Human Brain Health, School of Psychology, University of Birmingham, Birmingham, UK

**Keywords:** behavioral ecology, decision-making, complex environments, sensory processing, social interactions, cross-species

## Abstract

The study of foraging is central to a renewed interest in naturalistic behavior in neuroscience. Applying a foraging framework grounded in behavioral ecology has enabled probing of the mechanisms underlying cognitive processes such as decision-making within a more ecological context. Yet, foraging also involves myriad other aspects, including navigation of complex environments, sensory processing, and social interactions. Here, we first provide a brief overview of the neuroscience of foraging decisions, and then combine insights from behavioral ecology and neuroscience to review the role of these additional dimensions of foraging. We conclude by highlighting four opportunities for the continued development of foraging as an ethological framework for neuroscience: integrating normative and implementation-level models, developing new tools, enabling cross-species comparisons, and fostering interdisciplinary collaboration.

## Highlights

Foraging theory offers an ethological framework for studying complex behavior in neuroscience, bridging the gap between simplified tasks and unconstrained behaviors.
Optimal foraging theory has inspired behavioral tasks and computational models for understanding the neural basis of decision-making across species.
Research from behavioral ecology and neuroscience can be integrated to shed light on the mechanisms of other aspects of foraging behaviors, such as the role of complex environments, sensory signals, and social contexts.
Studying the brain through the lens of foraging allows neuroscientists to leverage advances in modeling, tool development, and cross-species research, while also benefiting from collaboration with ethologists and behavioral ecologists.

## Neuroscience through the lens of foraging

Neuroscience has classically employed a reductionist approach, combining controlled behavioral assays with the systematic manipulation of a small number of variables [1]. This paradigm has generated substantial insights into the neural basis of cognition and behavior. However, there are long-standing concerns about the generalizability of such findings beyond the laboratory [2,3], in part because the relationship of the brain to behavior is deeply degenerate [4,5]. Such concerns have prompted renewed interest in the adoption of ethologically inspired paradigms in neuroscience [2,3,6., 7., 8.].

**Figure 1:**
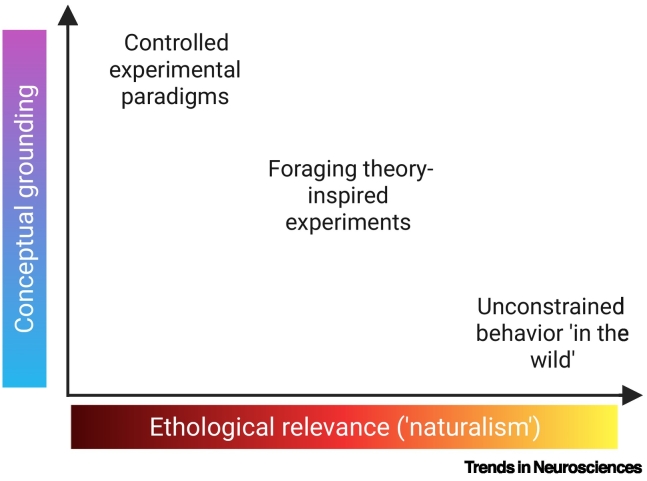
General classification of experimental paradigms in neuroscience in terms of their conceptual grounding and ethological relevance. Neuroscience experiments can be very broadly categorized along two axes: conceptual grounding (how strongly they are shaped by predictions from existing theory or models) and ethological relevance (how closely the behavior reflects the natural behavioral repertoire of the organism). Neuroscience studies informed by behavioral ecology theories of foraging can achieve a balance between the two through being inspired by theories of natural behavior. Figure created in BioRender. Grima, L. (2025) https://BioRender.com/llyrjja. This figure was inspired, in part, by Figure 1 in [12].

As part of this drive toward ‘naturalistic’ neuroscience, there has been a growing focus on the neuroscience of foraging. Foraging broadly encompasses the process by which organisms search for and exploit resources in their environment [9,10] (Box 1). Within behavioral ecology, foraging behaviors are frequently examined through the use of optimality models that predict ideal behavior under known environmental constraints [9., 10., 11.]. A foraging framework therefore provides a basis for addressing an increasing emphasis on behavioral complexity in neuroscience, while also serving as a theoretical foundation for predicting and interpreting both behavioral and neural data [12] (Figure 1).

Box 1 **Definitions of foraging**

Definitions of foraging vary widely across ethology and neuroscience, reflecting diverse theoretical perspectives and research focuses. Broadly, these definitions can be grouped into two classes: those that describe the general processes by which organisms search for and acquire resources, and those grounded in foraging theory – a central framework in behavioral ecology for modeling such behavior. The latter includes optimal foraging theory [9,11] and can be applied to cognitive domains, suggesting that similar mechanisms may underlie both resource foraging and the navigation of memory or knowledge spaces [138]. Other definitions within foraging theory propose that foraging is restricted to serial search on single options, thereby differentiating it from search and decision-making [17]. Neuroscience research has drawn on both classes of definitions, but most commonly frames the process in terms of neural mechanisms mediating the trade-off between exploitation of known options and exploration of new options [15].

Alt-text: Box 1

To date, foraging studies in neuroscience have primarily focused on the study of decision-making [13., 14., 15., 16.]. In natural settings, however, foraging encompasses a broader set of processes, including navigation through complex and structured environments, integration of sensory information, and social dynamics. We thus begin this review with a brief overview of insights from the neuroscience of foraging decisions, followed by a discussion of the broader literature from both behavioral ecology and neuroscience that addresses these aspects. For clarity, we organize these discussions around conventional cognitive and behavioral neuroscience domains, namely, navigation, sensory processing, and social cognition. However, we emphasize that the advantage of a foraging framework lies in its ability to integrate across these domains, given that in natural contexts these processes interact dynamically to shape behavior.

We finally highlight opportunities to extend the application of foraging-inspired frameworks in neuroscience. These include integrating normative and implementation-level models of behavior, advancing methodological tools, leveraging cross-species comparisons, and increasing collaboration between behavioral ecologists and neuroscientists. Building on this, we propose future directions to advance the use of foraging as a foundational ethological framework in neuroscience, moving beyond optimality models of decision-making and toward integration of diverse aspects of behavior and cognition.

## Foraging as decision-making

**Figure 2:**
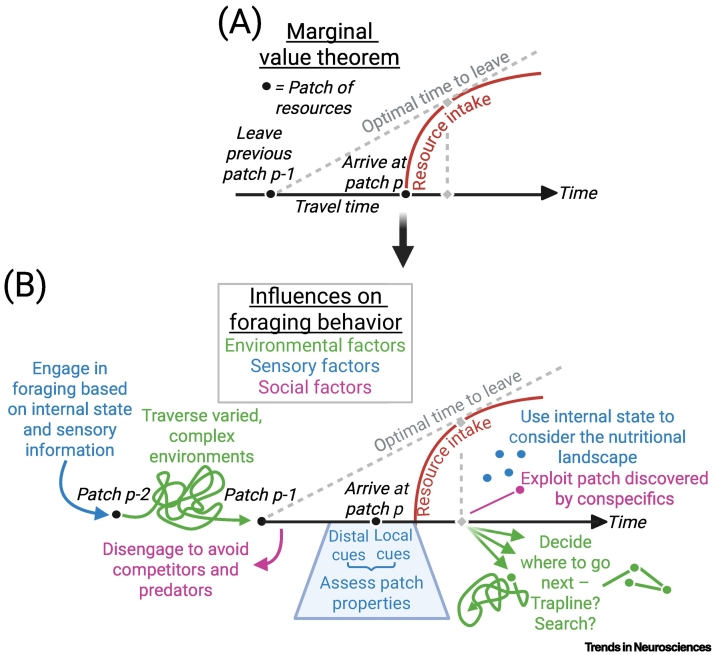
Illustration of core calculations of the marginal value theorem, and additional influences that can shape foraging behaviors beyond the marginal value theorem. (A) The marginal value theorem posits that the optimal time to leave a depleting patch of resources is when the rate of resource intake within the patch falls below the average rate of reward in the environment, including time spent traveling between patches. (B) Extending beyond the marginal value theorem, additional influences on foraging behavior include environmental, sensory, and social factors. The figure illustrates some examples of these aspects and the stages of foraging they influence beyond the decision to stay or leave a patch of resources. Figure created in BioRender. Grima, L. (2025) https://BioRender.com/qy5c2da.

A classical view from behavioral ecology frames foraging as a sequential decision-making process in which organisms repeatedly choose whether to pursue or abandon an encountered resource in favor of an alternative [11,17]. This perspective is most often applied to patch foraging, where resources are distributed discontinuously in the environment – a scenario that forms the primary focus of this review. Within this context, optimal foraging theory represents a class of normative optimality models that assume animals have evolved to maximize resource intake while minimizing cost [9]. A cornerstone of optimal foraging theory is the marginal value theorem, which applies this approach to environments where resources are monotonically depleted with consumption [11]. The marginal value theorem posits that the optimal time to leave a depleting resource patch is when its instantaneous rate of intake falls below the average rate of the environment (Figure 2A).

The marginal value theorem has been influential for its simplicity, intuitiveness, and apparent ubiquity; the behavior of a variety of non-human species in natural environments [18., 19., 20.] and in laboratory settings [21., 22., 23., 24.] has been found to align with its predictions. In humans, the marginal value theorem has been applied to characterize decisions made by hunter-gatherers [25], and to examine how decision-making evolves across learning [26] and over development [27]. These successful applications suggest that the marginal value theorem captures some fundamental features of decision-making in patchy environments, motivating recent efforts to identify the neural substrates of its decision variables.

One formulation of the marginal value theorem as an explicit computation draws on evidence accumulation models from perceptual decision-making [28], framing foraging decisions as a drift-diffusion process in which the forager leaves the current patch once the accumulated utility signal crosses a decision threshold [29]. Supporting this, neural activity in the dorsal anterior cingulate cortex of both human and non-human primates, as well as rodents, has been found to resemble drift-diffusion dynamics that encode the relative value of leaving a patch [22,30., 31., 32.], although the causal role of this signal remains debated [22,33]. Neuromodulatory systems have been more directly implicated in driving patch-leaving behavior: across mammals, increased serotonin, acetylcholine, and noradrenaline levels have been linked to sustained patch exploitation [34,35]. The role of dopamine in patch foraging may depend on the timescale of its signaling: slower, tonic fluctuations are thought to track the average environment reward rate [36,37], while faster, phasic signaling propagates value through space [38] or reflects the learning rate when many potential resources are available during foraging [39]. Similar functions have been attributed to these neuromodulators or their homologues in invertebrates [40., 41., 42., 43.].

However, the marginal value theorem and related optimal foraging theories were not developed to explain the mechanistic ‘how’ of behavior, but rather the evolutionary ‘why’ [44], serving as intuitive frameworks to identify adaptive solutions within highly idealized scenarios. While variables from the marginal value theorem have been successfully linked to neural activity, we argue that the simplifying assumptions underlying such optimality models – often carried over into laboratory settings – may narrow the scope of behaviors and mechanisms considered by neuroscientists. In the following sections, we discuss three additional factors that challenge such models (Figure 2B), examine their counterparts in behavioral ecology, and explore how neural processes might be studied to account for these complexities.

## Foraging in complex and structured environments

Optimality models of foraging decisions treat travel between resources as a temporal cost to be weighed against time spent gathering resources [9]. These models often assume simple, static environments that are fully known to the forager from the outset [45]. However, natural environments, with their diverse spatial and temporal features, pose significant behavioral and computational challenges, such as evaluating traversal effort and uncertainty [24], avoiding predators [46], and navigating terrain (Box 2). A comprehensive understanding of the mechanisms that give rise to foraging behaviors therefore, requires studying them under conditions that reflect naturally occurring challenges [2]. Indeed, behaviors that appear irrational in laboratory contexts can often be explained by the heterogeneous and autocorrelated nature of real environments [47].

Box 2 **Navigation and foraging**

Spatial navigation is the process by which an organism determines and maintains a course from one location to another using either external cues or internal representations [139]. Therefore, navigation can be understood as a distinct subcomponent of foraging such that effective navigation is integral to overall foraging success, particularly when the goal location is known. Conversely, studying neural computations underlying spatial orientation and navigation using foraging-inspired assays ensures a well-defined behavioral context. This permits control of the motivational factors driving spatial orientation and provides a framework in which to define and quantify successful navigation. One core navigational strategy, path integration-based homing, has been studied extensively in the context of foraging in a wide range of species [140]. The nature of the computational problem that path integration solves is reflected in the highly conserved neural circuitry thought to support it: a neural representation of head direction. Such head direction representations have been long identified in rodents, and more recently discovered in invertebrate systems [141]. While rapid progress has been made in identifying and characterizing this and other spatial representations in the brain using partially restrained animals and simple environments, current understanding of how these representations are used to ultimately guide behavior, especially in complex, naturalistic environments, is limited. This knowledge gap may be addressed by combining neural recording and perturbation techniques with foraging-inspired behavioral assays that shape the animal’s motivation and provide a clear framework for interpreting its actions.

Alt-text: Box 2

One such heterogeneous feature of natural environments is spatial inhomogeneity, in particular the discontinuous distribution of resources [9]. This characteristic inspired the framing of patch-leaving problems addressed by the marginal value theorem. However, this raises the question: what exactly constitutes a patch? While patches are more straightforward to define in experimental settings, natural landscapes rarely consist of discrete, consistently scaled distributions of resources [48], complicating the application of the marginal value theorem in real-world environments [26]. One approach to addressing this is to define patches on the basis of behavior. For example, area-restricted search captures how organisms adjust their search strategy during foraging in response to resource encounters, shifting from global exploration to local exploitation [49,50]. This can yield patch-leaving times consistent with marginal value theorem predictions without defining patch boundaries *a priori* [51], suggesting that internal representation of patches may not be strictly necessary for some foraging behaviors.

As mentioned previously, natural environments are also often autocorrelated – finding food in one location increases the likelihood of finding more nearby. Therefore, discovering efficient paths between different resources and uncovering their underlying structure is of benefit to most organisms. One example of multipatch foraging is traplining, where an individual executes repeated sequential visits to a series of feeding locations [52]. While most often studied in pollinator species, the prevalence and neural basis of this behavior have been explored across a broader range of organisms. For example, recordings from non-human primates have revealed that neural activity in the posterior cingulate cortex can represent the configuration of options when executing traplining [53].

Traplining and area-restricted search (or local search) are behaviors commonly observed across many species. However, research into their neural computations suggests diverse, redundant mechanisms rather than a small number of conserved principles [54]. This is intuitive; area-restricted search, for example, can be generated through a number of behavioral mechanisms, including path integration-mediated search [55] or search based on adaptation of locomotor variables alone [54]. Similarly, traplining can be generated through the development of simple heuristics [53] or more complex representations such as cognitive maps [56], although whether invertebrates in particular develop maplike representations is debated [57].

A final challenge of natural environments relates to the wide range of spatial scales over which foraging can unfold, far exceeding those of typical laboratory assays. For example, wandering albatrosses (*Diomedea exulans*) forage over distances spanning hundreds of meters to tens of thousands of kilometers, adjusting the tortuosity of their flight to the spatial scale of their journey [58]. To some extent, these spatial scales can be simulated in the laboratory by using virtual reality techniques, which allow for arbitrarily large environments and can be combined with high-yield neural recordings [59]. However, this approach often requires physical restraint of the animal, limiting its behavioral repertoire. Studies directly comparing behavior and neural activity in tethered and freely moving animals suggest that behavioral motifs and neural computations generalize to some degree [60], although certain dynamics may only be observable in freely moving contexts [61]. An alternative approach is to ‘bring the laboratory outside’ and study foraging behavior in natural settings. Doing so has uncovered entirely new behavioral and neural dynamics not observable in the laboratory. For example, bat hippocampal place cells recorded in very large environments can exhibit multiple place fields of widely varying scales in contrast to the typical single-field place cells observed in laboratory settings [62].

Together, these findings highlight that complex environments can shape behavioral strategies to account for the broader spatial and temporal challenges of foraging. This contrasts with typical trial-based laboratory studies in reduced environments that often do not maintain temporal contiguities and render space irrelevant or without structure [16]. Closer incorporation of these additional environmental complexities in future work is therefore necessary for advancing an integrated understanding of foraging behaviors and their relationship to neural dynamics.

## Sensory perception and internal drivers of foraging

As previously discussed, one successful modeling approach to decision-making during foraging is as a sensory evidence accumulation process [29]. In natural environments, however, sensory information is leveraged not just at the decision phase, but throughout foraging; from locating resources [63], assessing their multidimensional characteristics, and simultaneously evaluating whether predators or competitors are nearby [64]. Moreover, animals rely on different sensory information at different stages of foraging, such as vision at long distances, olfaction and hygrosensation at intermediate distances, and taste upon contact [65., 66., 67., 68.]. Indeed, foraging strategies can be directly shaped by the availability of such sensory cues, as well as an animal’s internal state [23,69].

During foraging, animals use diverse, often redundant, external stimuli to identify potential resources. For example, pollinators use odor, color, shape, local carbon dioxide (CO_2_), and moisture cues to select flowers [70]. Mosquitos similarly use multimodal cues to detect a blood meal with compensatory mechanisms that maintain foraging effectiveness even when one sensory modality is lost [71]. Beyond the multimodal nature of food cue sensing, natural sensory cues can be dynamic and complex; for example, plume tracking requires robust multisensory integration in turbulent environments [72] but can also adapt based on experience [73].

The perception of complex cues can be directly modulated by internal state; hunger enhances food cue responses in the postrhinal cortex in mice [74] and modulates chemosensory responses in *Caenorhabditis elegans* and *Drosophila* [75], suggesting the gating of sensory information according to physiological need. This influences not only foraging decisions [23], but also navigational strategies. For example, once foraging ants have learned fixed outbound and inbound routes between their nest and a resource, they rely on their inbound route memory only after finding food – in other words, when they are in a state of ‘wanting’ to return home [76]. This hunger-based internal state is mediated by brain–body interactions through neuropeptides, hormones, and neuromodulators across taxa; in mammals, hormones released from adipose tissue and neuromodulators from gastrointestinal tracts regulate satiety and food intake via neurons in the hypothalamus [77], and in *C. elegans*, bacteria that produce vitamin B_12_ in the gut reduces cholinergic signaling in the nervous system, affecting levels of turning behavior relevant to exploration [78].

Internal state itself is multidimensional, requiring the balancing of multiple nutrient needs to ensure the survival of the organism. Some animals develop nutrient-specific appetites, preferring protein-rich food when internal protein levels are low to achieve ‘nutrient homeostasis’ [79]. Therefore, nutrient states affect which resource cues animals will respond to, and what feeding decisions they will make when faced with multiple options [80]. This can also be inferred from the nutritional geometry framework, which allows quantification of optimal nutritional intake given resource constraints [81]. Other theoretical work has extended optimal foraging theory to account for this balancing of multiple nutrient needs by constructing a ‘fitness landscape’, where the optimal choice is one where minimal fitness cost is incurred [82]. Internal state can also incorporate moods and emotions, with recent evidence in humans revealing homeostatic interactions between stress and foraging behavior [83]. Finally, foraging organisms must evaluate these multidimensional needs against competing external sensory cues reflecting, for example, risk by predation. Evidence suggests that risky environments shape animals’ decisions to forage even when accounting for nutritional state [84].

Together, these findings underscore the important role of sensory processing, both internal and external, in all aspects of foraging behaviors. Sensory perception may, in fact, influence foraging success more than an organism’s movement strategy [85]. This presents challenges for mechanistic models of foraging behavior: for instance, whether unimodal evidence accumulation frameworks can generalize to multisensory contexts remains unclear. More broadly, sensory input and internal state jointly shape behavioral goals at any given moment, including disengaging with foraging entirely [86]. Therefore, studying foraging in more naturalistic contexts – with multimodal cues, competing demands, and varying risk – will enable advanced modeling of diverse aspects of foraging behavior and their underlying neural mechanisms.

## Social dimensions of foraging

Thus far, we have discussed the foraging of individual organisms. However, the behavioral ecology of social foraging, including how individuals forage for others [87], in the presence of others, and collectively [88], has also been extensively studied. By foraging together, animals can learn from others and share information about when, where, what, and how to eat [89]; reduce the risk of predation [90]; or engage in collective decision-making about when to leave patches or pursue prey [91].

Similar to the study of individual foraging decisions, optimal foraging theory has shaped task design and modeling in social neuroscience, often by applying the marginal value theorem to patch leaving in social contexts [92,93]. Similarly, evidence accumulation models have been used to understand how individuals share information about patch quality [94] or decide to leave social interactions [95]. However, despite the usefulness of such models, they often require modification to reflect the dynamics of real-world social strategies. For example, optimal foraging theory does not offer an account of how social variables or strategies for foraging, such as observing and learning from other foragers [96], inferring the intentions of others [97], and representation of social network structures [98], are encoded.

There are a number of behavioral ecology models that address multiorganism behavior. For example, the ideal free distribution theory predicts that foragers will distribute themselves among patches to equalize individual payoff and minimize competition, highlighting how social information can shape environment use [99]. The influence of competition on foraging has been explored at the neural level in mammals; the cingulate cortex encodes the value of patches in competitive contexts in humans [100] and drives competitive effort during foraging in mice [101]. Further, in humans, the amygdala has been shown to track the dynamics of competition density during foraging decisions [102]. In invertebrates, competition has been studied behaviorally; for example, sugar beet root aphid stem mothers typically distribute themselves across galling sites consistent with ideal free distribution principles [103]. Less is known, however, about the neural basis of competitive processes in invertebrates during foraging.

Producer-scrounger models further distinguish individuals who search for resources from those who exploit others’ finds [104]. This framework explains stable role distributions in the wild and offers insights into social dynamics and how they can be studied in neuroscience. For example, human virtual reality studies reveal increased scrounging in clustered-resource environments, where individuals join others at discovered patches [105]. In rodents, producer-scrounger roles have been modeled within a seminatural ‘micro-society’ as arising from individual traits and social constraints [106]. Here, the authors identified ventral tegmental area dopamine neurons as a neural substrate of this role differentiation; scrounging rodents showed increased dopaminergic activity when observing peers press a lever for food, a response not observed in producers.

Together, these findings point to several directions for advancing the understanding of social foraging and its neural basis within multiorganism contexts. Moreover, social foraging is closely intertwined with other key aspects of foraging behavior. For example, in clonal raider ants, internal states mediated by neuropeptides like inotocin regulate division of labor during collective foraging [107], highlighting the need to incorporate state dependence when studying neural substrates in cooperative species. Ultimately, research on social foraging offers a reciprocal opportunity: to decode the neural mechanisms underlying foraging strategies and to gain broader insight into the neural basis of social behavior across diverse species.

## Opportunities for the future of foraging research in neuroscience

**Figure 3:**
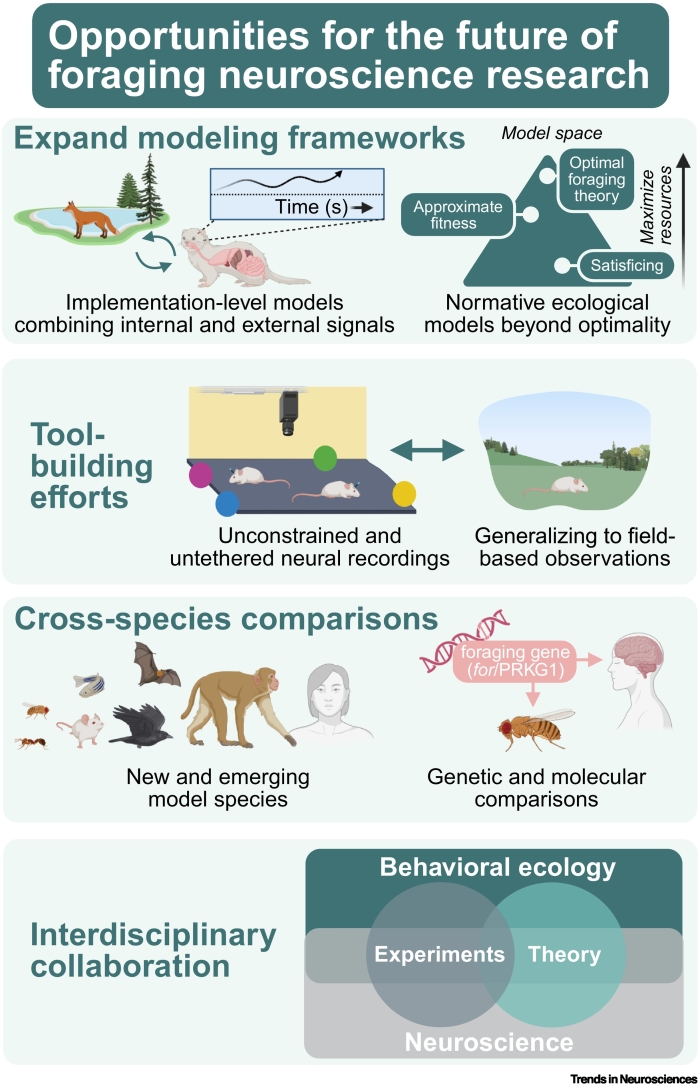
Opportunities for future neuroscience foraging research. An illustration of possible avenues for foraging research going forward, in particular, continued integration of implementation-level models from neuroscience and normative models from ecology, tool building to increase the complexity of environments being used and to generalize outside of the laboratory, cross-species comparisons, and interdisciplinary collaboration with the field of behavioral ecology. Figure created in BioRender. Grima, L. (2025). https://BioRender.com/gwvvwxi.

Neuroscientists have turned to foraging as a model, as a set of behaviors, and as an ethological framework within which to advance the mechanistic understanding of cognitive processes – in particular, of decision-making. Going forward, incorporating other aspects of foraging behaviors offers a promising path for broadening current understanding of diverse cognitive and behavioral processes and their interactions. Here, we discuss four emerging opportunities for progress in the field of foraging neuroscience (Figure 3).

### Embracing the complementary roles of normative and implementation-level models beyond optimality

Foraging behavior has become an attractive focus of research for neuroscientists, partly due to the predictive power of well-established descriptive and normative models from behavioral ecology [108]. The latter have been especially influential in neuroscience, and have been readily adapted into implementation-level models [109], for example, by extending the marginal value theorem to incorporate continual learning in Bayesian [110], dynamical [111], and reinforcement learning [112] frameworks. This has allowed neuroscientists to tap into possible biological mechanisms, identifying the encoding or representation of variables from such models in the brain [113].

Both ethologically driven normative models (what should be done to maximize utility) and implementation-level models (how such computations are realized algorithmically and in the brain) have strengths and limitations, as discussed elsewhere [108]. However, it has been suggested that each offers a distinct perspective for bridging levels of abstraction in the study of the neural basis of behavior [114]. Following others [13], we propose that foraging provides an ideal framework to leverage the complementary strengths of these approaches.

However, to date, the field of neuroscience has primarily approached foraging through the lens of optimality models. Yet the notion that animals operate as optimizing agents remains contested [45]. In natural environments, it is often unclear what should be maximized, as factors including those reviewed here – social, sensory, and environmental context – can dynamically shift an organism’s ‘objective function’ (i.e., the specific quantity the organism is optimizing through its behavior). Given this, we encourage neuroscientists to engage more broadly with the behavioral ecology literature beyond optimal foraging theory. For example, approximate fitness-seeking models preserve the idea of adaptive behavior while relaxing strict optimality assumptions, better reflecting real-world complexity [115]. Moreover, an alternative to optimal foraging theory is offered by ‘satisficing models’, which predict behavioral strategies aimed at meeting minimum acceptable thresholds rather than achieving an optimal outcome [116]. Inspiration from such behavioral ecology theories, alongside direct observation of behavior, may create opportunities to test models in neuroscience that would otherwise go unexplored given the assumption of optimality.

### Continued tool development for bringing the inside out, and the outside in

The growth of foraging neuroscience has been driven in part by the development of tools that permit the study of unconstrained behavior in tandem with neurophysiology. Deep learning-based techniques for the automated quantification of behavior now enable high-throughput yet fine-grained analysis of multiorganism continuous behavior [117], as well as its automatic segmentation into modules or ‘syllables’ [118,119]. Generative models developed from such tools can be applied to organisms both inside and outside the laboratory, offering opportunities for paradigms centered on closed-loop control of natural behaviors rather than relying on triggering of experimenter-controlled apparatuses [120].

A parallel approach centers on custom-designed apparatuses for freely moving behavior, enabling controlled yet multidimensional manipulation of the environment [121]. This has been used, for instance, to study how mice hunt crickets across many locations by controlling cricket placement, release, and the presentation of auditory stimuli [122], or to mimic predator–prey interactions between mice and robots during navigation [123].

With regard to physiology, it is now possible to record from large populations of neurons in untethered rodents, bats, birds, and non-human primates both in the laboratory [124., 125., 126.] and (in some cases) outdoors [62]. Untethered perturbations are also becoming increasingly achievable, for example, through the use of magnetogenetics in mice [127] or wearable transcranial magnetic stimulation devices in humans [128]. Data from studies using these new tools could reveal whether findings from the laboratory generalize to real-world observations, and inform model development to directly test assumptions of foraging models and the cognitive processes involved.

### Exploiting cross-species comparisons

It has been argued that the primary function of the brain is to produce adaptive behavior, particularly to acquire resources necessary for survival [129]. Therefore, foraging represents an ideal case study for cross-species investigation. For example, the ‘foraging (*for*)’ gene, first identified in *Drosophila* larvae and linked to their fixed ‘sitter’ and ‘rover’ foraging strategies [130], has been associated with search behaviors in humans and *C. elegans* through its ortholog [131,132]. A mechanistic example is the implementation of operant matching – a behavioral principle whereby an organism allocates its response proportional to the history of reward received at each option [133] – through synaptic plasticity [134]. Both mice [39] and *Drosophila* [41] have been shown to exhibit behavior consistent with the matching law during multioption foraging.

These observations suggest that such core computations may be evolutionarily retained, or appear through convergent evolution, and then adapted to fit varying ecological demands. Indeed, it has been argued that differences in foraging niches between rodents and primates have contributed to species-specific cortical specializations [135]. However, testing these hypotheses requires experiments tailored to the unique life history and environment of a given species, while retaining aspects that make the behavior under study comparable across species. A further challenge lies in the reliance of neuroscience on domesticated laboratory animals, whose behavior and ecology can differ substantially from their wild counterparts. Direct comparisons with wild populations offer an opportunity to discern which features of foraging are generalizable, and what aspects of behavior may be absent in laboratory settings [136].

### Integrating research fields, approaches, and conceptual foundations

While behavioral ecology and neuroscience have traditionally focused on different levels of analysis, foraging offers a natural point of convergence through which the domains of decision-making, spatial navigation, multisensory integration, and social behavior, among others, can be studied. This aligns with increasingly integrative perspectives on brain function; rather than viewing different cognitive or behavioral processes as isolated to specialized brain regions, current thinking emphasizes that these processes often involve the interaction of multiple brain areas, framing the brain as a cohesive system for adaptive behavior [137].

To deepen current understanding of the neural control of behavior in ecological contexts, neuroscientists should increasingly adopt naturalistic paradigms that probe multiple processes simultaneously [6,12]. Achieving this while preserving conceptual clarity will require active collaboration with ethologists and behavioral ecologists, whose expertise can be leveraged to further refine behavioral paradigms, identify ecologically relevant variables, and guide the interpretation of neural data within a meaningful ecological framework.

## Concluding remarks

Foraging offers a framework for examining brain function in complex, real-world contexts grounded by theory. While canonical behavioral ecology models of foraging have provided valuable insights, this review underscores the advantage of an increasingly multidimensional approach that accounts for the dynamic, multiscale nature of foraging. This includes moving beyond simple decision-making paradigms to incorporate the environmental, sensory, and social complexities that shape foraging behaviors.

Emerging technologies such as virtual reality, advanced neural recording techniques, and computational modeling are being incorporated into foraging research, offering more integrative perspectives on how the brain supports complex behavior across species. Moving forward, research will benefit from cross-disciplinary collaboration that brings together neuroscience, ethology, computational modeling, and behavioral ecology. These integrative approaches are well-positioned to address questions that span species and levels of analysis (see Outstanding questions). While focused on foraging, such efforts can contribute to broader insights into the neural basis of behavior. By engaging with the complexity of foraging, researchers may identify novel principles that support adaptive behavior and its neural underpinnings.

Outstanding questions What are the core neural computations underlying foraging that are conserved across species, and how are these adapted to specific ecological niches?

How can theoretical frameworks be updated to bridge the evolutionary purpose and the neural implementation of foraging behavior?

How can current computational frameworks be extended to provide a unifying account of how multiple, sometimes conflicting, sensory inputs are processed to guide foraging behaviors?

How can automated behavioral analysis tools help to define and quantify ‘decision points’ in continuous, naturalistic foraging behavior?

To what extent are inferences about neural dynamics in head-fixed foraging tasks applicable to freely moving foraging behavior, and is this dependent on species?

What roles do genetic and molecular processes play in shaping individual differences in foraging strategies across species?

What does it mean for a foraging-inspired experiment to be ‘ethological enough’, and does this vary by species or neural function?

Research into the mechanistic basis of foraging would benefit from interdisciplinary collaborations between theorists, experimentalists, ethologists, and ecologists. Which approaches could be used for motivating effective collaboration among these domains to bridge their diverse perspectives?

Alt-text: Outstanding questions

## Author contributions

All authors conceived of, wrote, and edited the review. Author order was determined alphabetically.
